# An optimized, robust and reproducible protocol to generate well-differentiated primary nasal epithelial models from extremely premature infants

**DOI:** 10.1038/s41598-019-56737-9

**Published:** 2019-12-27

**Authors:** Anke Martens, Gabriele Amann, Katy Schmidt, René Gaupmann, Bianca Böhm, Eleonora Dehlink, Zsolt Szépfalusi, Elisabeth Förster-Waldl, Angelika Berger, Nanna Fyhrquist, Harri Alenius, Lukas Wisgrill

**Affiliations:** 1 Division of Neonatology, Pediatric Intensive Care & Neuropediatrics, Comprehensive Center for Pediatrics, Medical University of Vienna, Vienna, Austria; 2 Institute of Clinical Pathology, Medical University of Vienna, Vienna, Austria; 3 Center for Anatomy and Cell Biology, Medical University of Vienna, Vienna, Austria; 4 Division of Pediatric Pulmonology, Allergology and Endocrinology, Comprehensive Center for Pediatrics, Medical University of Vienna, Vienna, Austria; 5 Center for Congenital Immunodeficiencies, Medical University of Vienna, Vienna, Austria; 6 Department of Bacteriology and Immunology, University of Helsinki, Helsinki, Finland; 7 Institute of Environmental Medicine, Karolinska Institutet, Stockholm, Sweden

**Keywords:** Experimental models of disease, Paediatric research

## Abstract

Extremely premature infants are prone to severe respiratory infections, and the mechanisms underlying this exceptional susceptibility are largely unknown. Nasal epithelial cells (NEC) represent the first-line of defense and adult-derived ALI cell culture models show promising results in mimicking *in vivo* physiology. Therefore, the aim of this study was to develop a robust and reliable protocol for generating well-differentiated cell culture models from NECs of extremely premature infants. Nasal brushing was performed in 13 extremely premature infants at term corrected age and in 11 healthy adult controls to obtain NECs for differentiation at air-liquid interface (ALI). Differentiation was verified using imaging and functional analysis. Successful isolation and differentiation was achieved for 5 (38.5%) preterm and 5 (45.5%) adult samples. Preterm and adult ALI-cultures both showed well-differentiated morphology and ciliary function, however, preterm cultures required significantly longer cultivation times for acquiring full differentiation (44 ± 3.92 vs. 23 ± 1.83 days; p < 0.0001). Moreover, we observed that recent respiratory support may impair successful NECs isolation. Herewithin, we describe a safe, reliable and reproducible method to generate well-differentiated ALI-models from NECs of extremely premature infants. These models provide a valuable foundation for further studies regarding immunological and inflammatory responses and respiratory disorders in extremely premature infants.

## Introduction

Viral infections affect young children worldwide leading to high hospitalization rates, increased health-care burden and even death among chronically ill patients^1,2^. The nasal airway epithelium is the first line of defense against invading viruses (e.g. respiratory syncytial virus), representing the “gatekeeper” against the progression towards severe lower airway tract infections^3^. Some patient cohorts, including extremely premature infants, exhibit a higher susceptibility towards respiratory infections and increased severity compared to the general population. Epithelial cell function, pathogen recognition as well as cellular pathways appear to be skewed towards diminished and/or polarized host immune responses leaving premature infants prone to viral infections^1,4,5^. In the emerging field of tailored and personalized medicine, patient-derived *in vitro* models are of very high value to pin-down individual physiological and pathophysiological processes in order to potentially unravel novel mechanisms or therapeutic targets.

In recent years, establishment of *in vitro* models from primary nasal epithelial cells (pNECs), which can be easily obtained by nasal brushing, opened up new possibilities to study the mucosal immune response of the nose^6^. This minimally invasive approach is especially suitable in the field of neonatal and pediatric research, allowing safe and ethical acquisition of pNECs from highly vulnerable patient cohorts such as extremely premature infants. Recent studies indicate the safety and feasibility to isolate and culture pNECs from term as well as moderate and late preterm infants^7,8^.

To date, a major obstacle in this new field of research is the lack of comprehensive protocols to isolate, propagate and differentiate pNECs of infants at air-liquid interface (ALI). Thus, the overarching aim of this study was to generate a robust and reproducible protocol for differentiating pNECs from premature infants at ALI. Using our adapted and optimized protocol, we were able to create, and comprehensively validate, fully-differentiated pNEC culture models from adults and extremely premature infants at term-corrected age.

## Results

### Patient characteristics, success rate and culture time

In total, 11 adult controls and 13 extremely premature infants at term corrected age were included in the study. The patient characteristics are summarized in Table 1. Nasal brush biopsies were very well tolerated by all participants. Only in two adult samples and one preterm sample minimal traces of blood were visible, tough neither actual epistaxis nor other adverse events occurred. The success rate of isolating pNEC was 7/11 in adult samples (63.6%) and 5/13 in preterm samples (38.5%), respectively. Due to the low isolation success rate in extremely premature infants, the impact of further clinical respiratory parameters was assessed (Table 2). It appears that in some infants, although the brushing was performed adequately, the recovery of pNECs was diminished. Interestingly, the days between sampling and the last day of respiratory support showed a trend towards statistical significance on the isolation success rate (p = 0.09), reducing the success rate if sampling was performed soon after or during respiratory support. One preterm pNEC culture was contaminated by a gentamicin-resistant *Escherichia coli*. Data are summarized in Table 2.

**Table 1:** Patient characteristics. Data are presented as mean ± standard deviation or as frequency (n (%)). n.a. = not applicable; PROM = premature rupture of membranes; BPD = bronchopulmonary dysplasia.

	Adults (n = 11)	Preterm (n = 13)
Age at sampling	28.1 ± 5.3 years	38.9 ± 1.5 weeks
Birth weight	n.a.	674 ± 105 grams
Gestational age	n.a.	25.2 ± 1.1 weeks
Weight at sampling	n.a.	3047 ± 630 grams
Caesarean section	n.a.	12 (92.3%)
Antenatal corticosteroids	n.a.	13 (100%)
PROM	n.a.	5 (38.4%)
BPD	n.a.	4 (30.8%)

**Table 2:** Clinical and respiratory data of successful and failed pNEC isolations in premature infants.

Parameter	pNEC – Success (n = 5)	pNEC – Failure (n = 8)	*p- value*
Gestational age (wks)	25.1 ± 0.7	25.3 ± 1.2	0.32
Birth weight (grams)	694 ± 124	664 ± 91	0.64
Age at sampling (wks)	39.2 ± 1.8	38.8 ± 1.3	0.45
Weight at sampling (grams)	3010 ± 729	3067 ± 567	0.89
BPD	1 (20%)	3 (37.5%)	1.00
respiratory support during sampling	0 (0%)	2 (25%)	0.48
Days between discontinuation of respiratory support and sampling day	29.2 ± 5.0	17.3 ± 12.8	0.09

Data are presented as mean ± standard deviation or as frequency (n (%)). wks = weeks; BPD = bronchopulmonary dysplasia.

**Figure 1:**
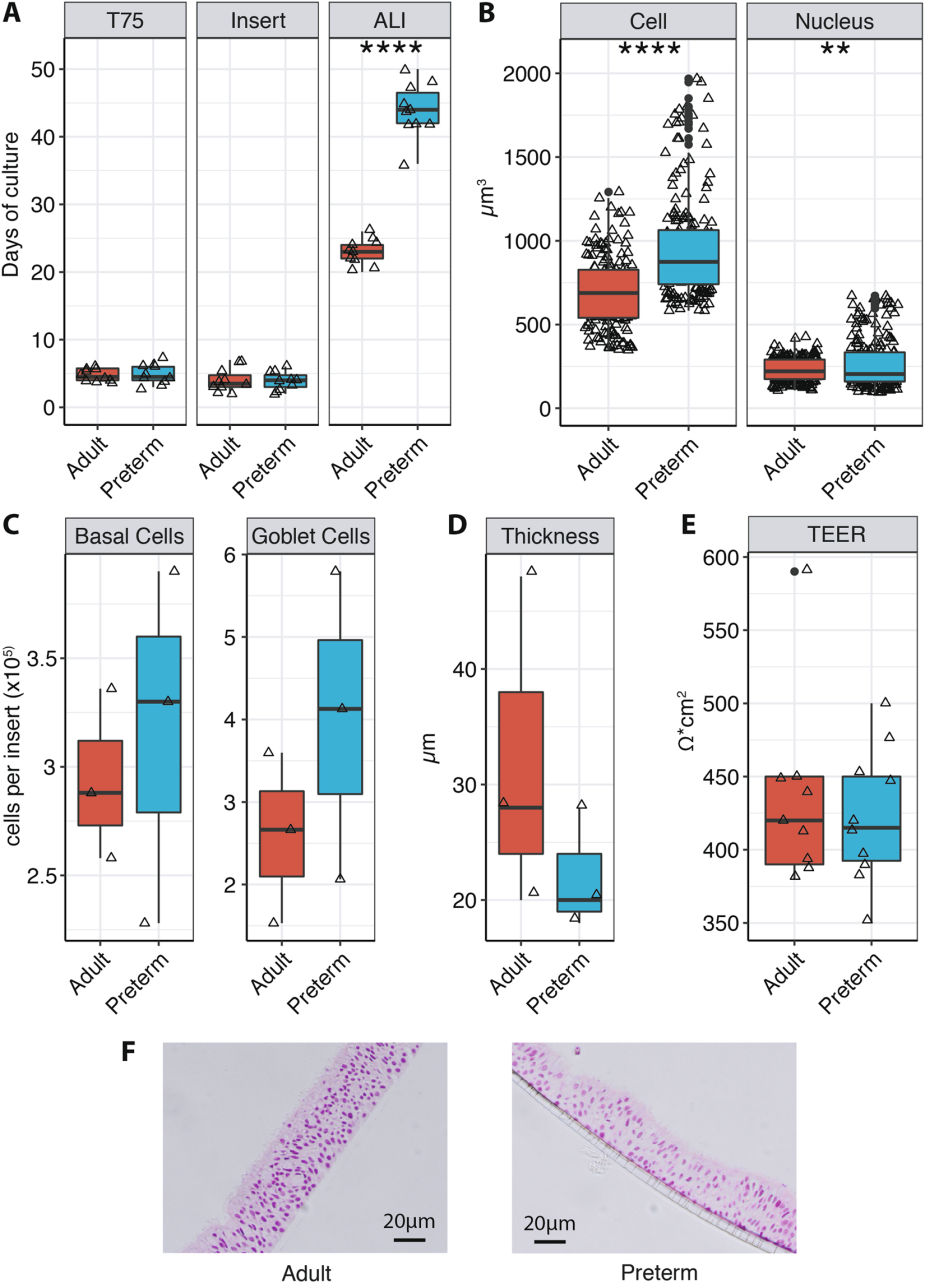
Times and metrics of ALI cultures. (**A**) Days in cultures needed to reach 70–80% confluency in T75 flasks, 100% confluency on transwell inserts and full differentiation at ALI for adult (red; n = 5) and preterm (green, n = 5) pNECs. Data from two independent experiments of each proband are shown. (**B**) Cell and nucleus volumes were calculated from 3D reconstructed confocal images using Imaris imaging software. Based on the in-build “Cells” algorithm, the cellular volumes were calculated for each identified cell based on nuclear (DAPI) and actin/plasma membrane (Phalloidin) staining. Data are summarized from four independent experiments. (**C**) The number of basal cells, goblet cells and (**D**) epithelial thickness was measured from confocal images of adults (n = 3) and preterm infants (n = 3) using the Imaris imaging software. (**E**) Transepithelial electrical resistance (TEER) from fully differentiated cell culture inserts was assessed for adult (n = 5) and preterm (n = 5) pNECs. Data from two independent experiments of each proband are shown. (**F**) H&E stained sections of adult (left panel) and preterm (right panel) ALI cultures. (**A**–**E**) Boxes represent the 25^th^ to 75^th^ percentile and error bars indicate the 5^th^ and the 95^th^ percentile. Median values are represented by the box middle line. Black dots indicate outliers. Black triangles represent each individual data point. Normal distribution was determined with Shapiro-Wilk-test. Normal distributed data was analyzed using student’s t-test and not-normal distributed data was analyzed using Mann-Whitney test. **p < 0.01, ****p < 0.0001.

A successful airlift was performed in 5/7 adult cases (71.4%) and in 5/5 preterm cases (100%). The pNECs of the failing adult samples did not grow to full confluency or showed leakage after several days at ALI (three independent attempts each). Adult and preterm pNECs displayed similar culture times to reach 70–80% confluency in the T75 flask (4.8 ± 0.919 vs. 4.8 ± 1.4 days) and 100% confluency in the transwell insert (4 ± 1.83 vs. 3.8 ± 1.32 days), respectively. However, preterm pNECs needed a significantly longer duration to exhibit signs of full differentiation compared to adult samples (44 ± 3.92 vs. 23 ± 1.83 days; p < 0.0001). ALI-differentiation was defined as uniformly distributed and intact cellular layer with cilial coverage and movement as well as mucous production in all four insert quadrants, as confirmed by light microscopy. Subjectively, preterm pNECs visually and light microscopically displayed a higher mucus production compared to adult controls and in immunofluorescence they showed a slightly higher, but not statistically significant, goblet cell count. Additionally, TEER was used to assess epithelial integrity showing comparable results between the groups. Furthermore, preterm and adult ALI cultures showed similar epithelial thickness (Fig. 1).

### Preterm and adult pNECs display fully-differentiated and comparable morphology

ALI cultures were checked at media change and showed similar morphology over time. Light microscopy movies of ciliary beating are provided in the Supplementary Files (Supplementary Video 1). To assess full differentiation of cultures in morphology and function, immunofluorescence microscopy, high-speed video microscopy analysis and TEER measurements were conducted.

**Figure 2:**
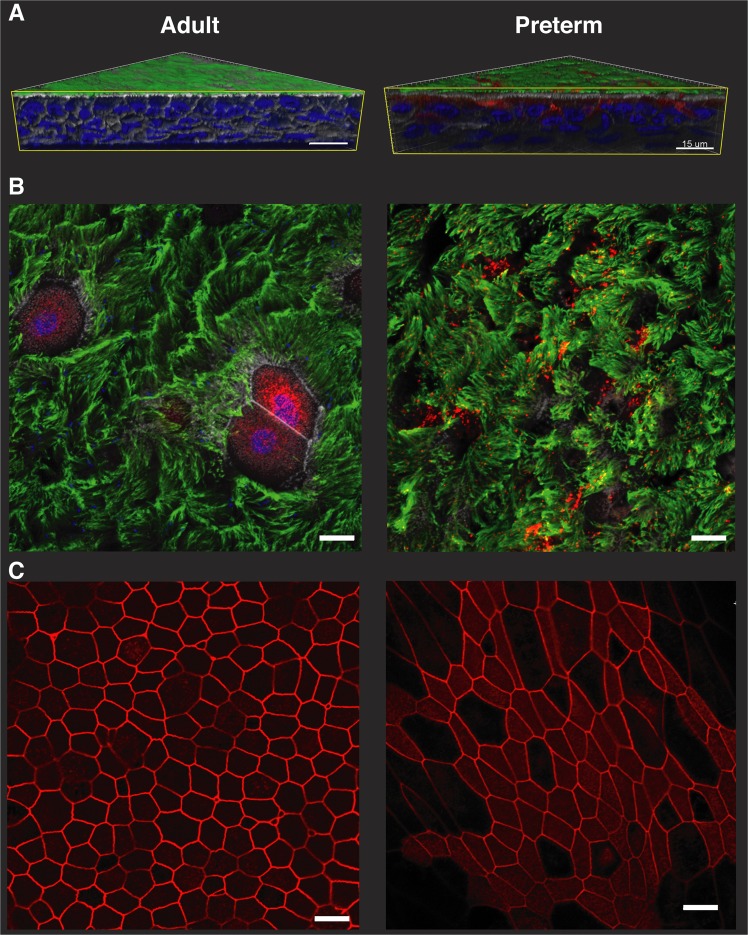
Comparison of culture morphology at ALI using confocal microscopy. (**A**) Fully differentiated ALI cultures from adults (n = 3, left panel) and extremely premature infants (n = 3, right panel) were assessed for differentiation and stratification using immunefluorescence. Representative 3D reconstructions from adult and preterm models (green = α-tubulin, blue = DAPI, white = Phalloidin, red = MUC5A). Scale bars: 15 µm. (**B**) Plain view with 63x magnification on the apical cell culture side depicting cilia (green = α-tubulin) and goblet cells (red = MUC5A). Nuclei and actin were counterstained with DAPI and Phalloidin, respectively. (**C**) Tight junctions were visualized utilizing ZO-1 staining (red). In (**B**,**C**), scale bars indicate 10 µm. All confocal images were obtained with an LSM700 confocal microscopy (Carl Zeiss) and images were processed using the Imaris imaging software (Bitplane).

**Figure 3:**
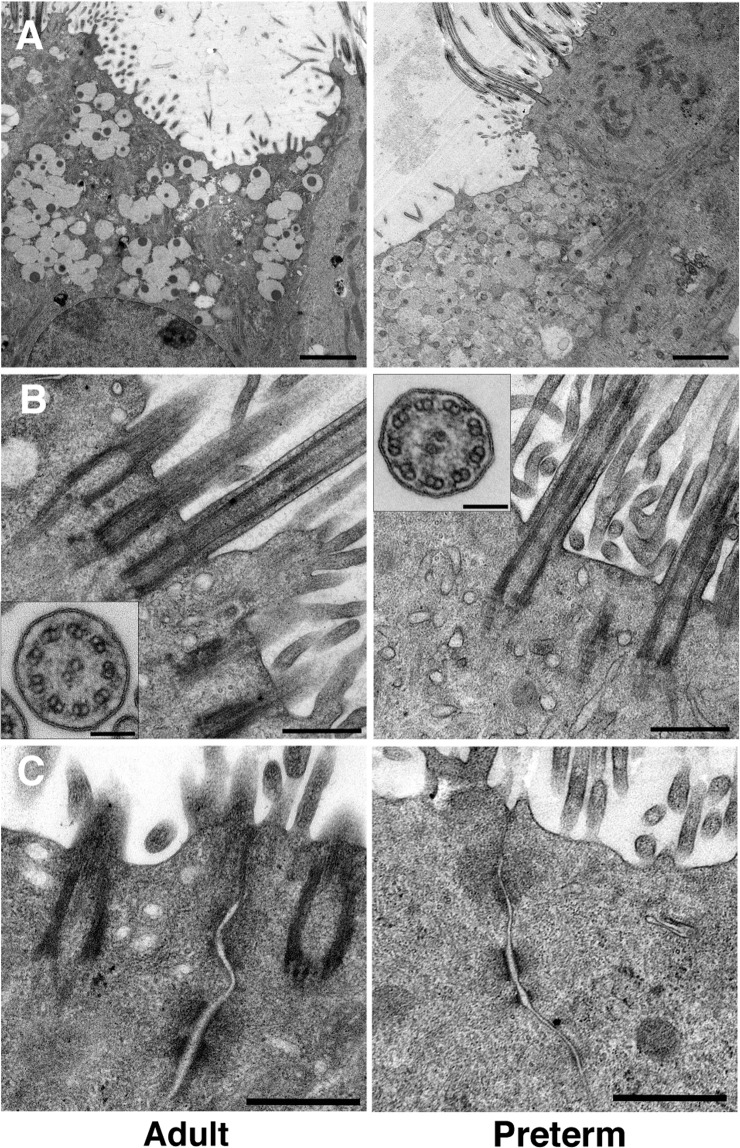
Ultrastructural assessment of ALI cultures using transmission electron microscopy. Fully differentiated ALI cultures from healthy adults (n = 3; left panel) and preterm infants (n = 3; right panel) were assessed for ultrastructural properties of goblet cells (**A**; scale bar = 2 µm), cilia (**B**; scale bar = 500 nm; scale bar inset = 100 nm) and tight junction complexes (**C**; scale bar = 500 nm). Representative images are shown. Pictures were acquired with a FEI Tecnai20 electron microscope and processed using Adobe Photoshop.

Adult and preterm ALI cultures displayed comparable differentiation and epithelial stratification as well as staining of cellular markers (Fig. 1F/Fig. 2). In the reconstructed 3D model, both groups exhibited multilayer stratification containing ciliated cells (α-tubulin+ cilia), goblet cells (MUC5A+ apical cells, Fig. 2A,B), firm tight junctions (ZO-1 staining, Fig. 2C) and basal cells (p63+ basal cells, basal cell count Fig. 1C, Supplementary Fig. 1). Next, transmission electron microscopy was used to assess ultrastructural properties of ALI cultures. Cultures from both groups displayed goblet cells as well as ciliated cells (Fig. 3A,B), as seen in the immunofluorescence staining before. Cross-sections of the cilia showed a typical ultrastructure containing central singlet microtubules, outer doublet microtubules, radial spokes and dynein arms (Fig. 3B, insets). Junctional complexes are represented in Fig. 3C.

### Functional ciliary analysis revealed comparable beat pattern in adult and preterm pNECs

In addition to morphological differentiation, the cell culture models were examined in terms of function. To assess the functionality of the cilia, ciliary movement pattern was analyzed using high-speed video microscopy. All analyzed samples showed normal cilial length and configuration as observed in immunofluorescence and ultrastructural analysis. Adult (n = 4) and preterm (n = 3) samples displayed comparable beat pattern and frequencies (11.2 ± 1.6 vs. 10.7 ± 1.0; p = 0.66), consistent with healthy ciliary beat frequencies in the literature^9^. Representative movies of the analysis are provided in the Supplementary Files (Supplementary Video 2).

## Discussion

We present a method to reliably generate well-differentiated cell culture models from pNECs of extremely premature infants, which mimic the nasal epithelium *in vivo*. Using different approaches, we showed successful differentiation in morphology and function: Our models exhibited the characteristics of the respiratory epithelium, including ciliated cells with regular beat patterns, mucus producing goblet cells, basal cells and junctional complexes. To our knowledge, this is the first study providing a comprehensive protocol for the isolation, propagation and differentiation of pNECs from extremely premature infants at term-corrected age.

There are different methods to obtain pNECs from probands. Depending on the cohort, intraoperative biopsies as well as scraping with cuvettes or brushes were used to acquire pNECs^6–8,10^ with brushing seemingly being the collection type of choice in infants showing high success rates with good safety profiles^7,8^. Hence, for our patient cohort, nasal brushing was considered to be the best suitable and minimally invasive option and thus ethically adequate, all the more as this technique is routinely performed in our hospital to collect nasal samples from infants and children for assessment of primary ciliary dyskinesia.

Prior to differentiating the pNECs on ALI our cells were cryopreserved and stored in liquid nitrogen. We show that our cell culture models mimic the nasal epithelium *in vivo*, hence cryopreservation seems not to alter morphology and function. Our findings are consistent with previous published data^8,11^. For instance, Groves *et al*. showed similar ALI differentiation times, morphology, TEER values and proportions of goblet and ciliated cells for infant pNECs cultured after or without cryopreservation^8^. These findings suggest, that cryopreserved pNECs provide a convenient way to reliably generate well-differentiated *in vitro* cell culture models for further research into the respiratory system of premature infants.

To date, only few studies address the cultivation of pNECs from term and moderate or late preterm infants. Miller, *et al*.^7^ assessed the differentiation of pNECs obtained from healthy neonates (>36 weeks gestational age) within 48 hours after birth. They isolated and propagated pNECs under submerged conditions up to passage 3 using a protocol which is comparable to ours. But they did not further differentiate the pNECs at ALI. The morphology of the cell cultures was assessed using phase contrast microscopy, electron microscopy and immunostaining for specific proteins of the epithelium (cytokeratin 19 and 13). The cell cultures showed some characteristics of epithelial cells, although e.g. junctional complexes were absent. This indicated that junctional complexes as we show here, first develop during the differentiation process at ALI. To study immunological processes or biochemical pathways of the nasal epithelium it is important to mimic the tissue *in vitro* as precisely as possible to the *in-vivo* situation. Thus, ALI cultures seem to be the method of choice.

Groves *et al*. differentiated the pNECs of term and preterm infants (>28 weeks gestational age) right after birth and one year later. They confirmed differentiation using light microscopy, immunofluorescence and TEER-measurements, which showed comparable results to ours. Functional aspects, as ciliary beat frequency, were not assessed. Interestingly, in this study, culture duration to achieve a fully differentiated phenotype was even longer in term (median 77.5 days; range 74–94 days) and preterm (median 80 days; range 61–133 days) infants compared to our results^8^. One potential source of this significant culture time difference might be due to the choice of culture medium. For our study, we decided to use Pneumacult-Ex Plus and -ALI medium from Stemcell Technologies based on easy-to-use preformulated supplementation, long-stability and recommendations from experienced colleagues working with adult samples. To our knowledge, data concerning the impact of different media formulations on growth and differentiation of nasal epithelium are missing. Although, a recent study has investigated this topic for normal human bronchial epithelial (NHBE) cells^12^. Rayner *et al*. found that different cell culture media impact various parameters in cell growth or differentiation processes, as cell density, cluster formation or the number of possible passages. Interestingly, the differentiation grade and the epithelial thickness differed evidently after 4 weeks at ALI, while they further observed that the duration to generate well-differentiated cell culture models depended on the medium used. According to Rayner *et al*. Pneumacult-Ex Plus and -ALI medium from STEMCELL Technologies are most suitable for successful differentiation of NHBE cells at ALI among the media tested. These results might apply for pNECs in a similar way and could explain the large difference between the cell culture time of Groves *et al*. and ours. Therefore, we strongly suggest that medium composition might influence differentiation times, morphology and function of pNECs.

In our study we observed lower isolation success rates for extremely premature infants compared to adults. Since our focus is in extremely premature infants, especially those with pulmonary comorbidities such as bronchopulmonary dysplasia, we looked for influencing factors on the isolation success rate, such as ongoing respiratory support. The time elapsed between discontinuation of respiratory support (nasal cannula) and sampling day appeared to influence the success rate of pNEC isolation (Table 2). Continuous flow and/or oxygen supplementation might harm the integrity of the epithelium and induce epithelial stress responses leading to excess cell death during the isolation procedure. However, this hypothesis needs to be tested in a larger cohort of extremely premature infants.

We were able to show that our protocol leads to well-differentiated and functional nasal epithelium with comparable tissue integrity, thickness, mucus production, cilial coverage and function between the adult and preterm group. Interestingly, we observed larger cells and nuclei in premature cultures compared to adults. The significance of these findings is yet unknown and, to our knowledge, has not been described elsewhere. Additionally, we visually detected a subjectively higher mucus production in cultures from premature infants and found slightly higher, but not statistically significant, goblet cell counts in premature infants compared to adults. This is in line with other studies showing higher goblet cell counts in nasal cells from infants^8,13^. This result might have implications for the pathogenesis of viral infections as it could provide an explanation for the increased risk of mucus plug formation and raised disease severity^14,15^.

Besides studying the mucosal immunity of the nose, our models might as well be useful to investigate diseases and immune responses of the lower respiratory tract. As for ethical and practical reasons, bronchoscopy and other methods to sample bronchial epithelial cells (BEC) can hardly be implemented in as vulnerable groups as premature infants. To date, there is an ongoing discussion if it is possible to use NECs as surrogates for BECs. A number of studies indicate a good correlation between NECs and BECs in terms of basal gene expression, cytokine secretion and/or receptor expression^13,16–19^. Morphologically, NEC and BEC cell cultures are comparable, however, the proportions of ciliated and goblet cells differ^13,20^. But immunological responses after viral infections could present differently in both groups^13,19^. Taken together, BECs appear to remain the ideal approach to study the lower respiratory tract. However, NECs serve as a suitable compromise to address these research questions in premature infants, whereby possible deviations from the actual *in vivo* situation must be considered when interpreting the results.

As different protocols are likely to have an impact on the differentiation process – and thus also on down-stream experiments and interpretation of results – comprehensive protocols are needed to ensure comparability within experimental settings as well as between different studies. We demonstrate that pNECs collection by nasal brushing has no adverse side effects in extremely premature infants. Taken together we provide a detailed, optimized, robust and comparatively fast protocol to generate well-differentiated pNEC models allowing safe, ethical and state-of-the-art *in vitro* assays to study respiratory diseases and mucosal immunological responses in the prone population of preterm infants.

## Methods

### Study population

Extremely premature infants (n = 13; birth weight < 1000 g; gestational age < 28 weeks) and healthy adult controls (n = 11, age between 23–40 years) were recruited at the Division of Neonatology of the Medical University of Vienna. Patient characteristics are summarized in Tables 1 and 2. Nasal brush biopsies of preterm infants were obtained at a term corrected age above 37 gestational weeks, to ensure comparability to full-term infants. Infants with known maternal autoimmune or immunological diseases, genetic disorders or congenital malformations at the time of recruitment were excluded. Only healthy, non-smoking and non-allergic adults without respiratory infection for four weeks before brushing were included. Adult probands had no history of premature birth. The study was approved by the ethics committee of the Medical University in Vienna (EK 2164/2017) and parents as well as adult participants gave informed written consent prior to study inclusion in accordance to the declaration of Helsinki.

### Nasal brush biopsy and sample preparation

The protocols for the differentiation of pNECs at ALI are adapted versions based on the protocol from Muller *et al*.^10^. Detailed protocols of the described procedures are attached as Supplementary Files (Supplement File 1). Nasal brush biopsies were performed to obtain pNECs. A moistened cytobrush (Cooper Surgical, Trumbull, Connecticut, USA) was inserted into the nostril and rotated gently several times. A new cytobrush was used for each nostril. Next, both brushes were transferred into a 15 ml conical tube, containing 8 ml RPMI 1640 medium (Gibco, Thermo Fisher Scientific, Waltham, Massachusetts, USA) supplemented with 1% Antibiotic-Antimycotic (100X, Gibco, Thermo Fisher Scientific) and 0.1% Gentamicin (50 mg/ml, Gibco, Thermo Fisher Scientific) and mucus and cells were detached by gently swirling from the brushes. The cell suspension was centrifuged, resuspended in Pneumacult-Ex Plus medium (Ex-P; Stemcell Technologies, Vancouver, CAN) and treated for 20 min with DNase I (1.5 mg/ml; Sigma Aldrich, St. Louis, Missouri, USA). Afterwards, the cell suspension was centrifuged, resuspended in Ex-P and seeded onto coated 12-well plates (coating buffer containing fibronectin (1 mg/ml, Gibco, Thermo Fisher), bovine serum albumin fraction V (BSA; 1 mg/ml; Sigma), PureCol collagen (1:100, AdvancedBioMatrix, San Diego, CA) in PBS without Ca^2+^/Mg^2+^)^21^.

### Passaging of pNECs

After initial expansion on 12-well plates, pNECs were detached with trypsin-EDTA solution (Sigma Aldrich) and seeded in coated T25 flasks (passage 1). When reaching 70–80% confluency, the cells were further propagated in coated T75 flasks (passage 2). After passage 2, the pNECs were frozen in serum-free BAMBANKER freezing medium (GC Lymphotec, Kyoto, JAP) and stored in liquid nitrogen until ALI-differentiation.

### ALI-Differentiation

For the ALI cultures, collagen-coated 12 mm Transwell inserts (Corning, USA) with a pore size of 0.4 µm and a transparent PET membrane were used. 300 000–500 000 cells were seeded per insert and maintained under submerged conditions in Ex-P medium. Once the cell layer was completely confluent, the ALI was established by aspirating the medium from the apical chamber and the medium in the basal chamber was switched to Pneumacult ALI (PC-ALI, Stemcell Technologies). ALI conditions were maintained until full differentiation was achieved. Full differentiation was defined by obvious mucus production, clearly visible ciliary beating throughout the insert and a transepithelial electrical resistance (TEER) > 300 Ωcm^2^.

### Immunofluorescence microscopy and image analysis

Inserts were fixed for 15 min at room temperature (RT) with 4% paraformaldehyde (PFA) and then washed three times with phosphate-buffered saline (PBS, Gibco, Thermo Fisher Scientific). Membranes were cut out and placed in a 24-well plate with 400 μl blocking buffer (PBS containing 5% bovine serum albumin (BSA, Sigma Aldrich) and 0.3% Triton X-100 (Sigma Aldrich)) for one hour at RT. Primary antibodies were diluted in staining buffer (PBS containing 1% BSA and 0.3% Triton X-100) and incubated overnight at 4 °C. The following primary antibodies were used: mouse anti-alpha tubulin (1:1000, ab24610, Abcam, Cambridge, UK), rabbit anti-Mucin 5AC (1:250, ab198294, Abcam), rabbit anti-ZO-1 (1:125, 40–2200, Invitrogen, Thermo Fisher Scientific) and rabbit anti-p63 (1:60, ab124762, Abcam). Subsequently, membranes were rinsed three times with 400 μl PBS and incubated with secondary antibodies for two hours at room temperature (goat anti-mouse Alexa Fluor 488 (1 μg/ml, A-11017) and goat anti-rabbit Alexa Fluor 546 (4 μg/ml, A-11071, both Invitrogen, Thermo Fisher Scientific) diluted in staining buffer). Membranes were washed three times with PBS and incubated with Phalloidin (1:40, A22287, Invitrogen, Thermo Fisher) for one hour at RT, followed by another washing step. Finally, membranes were stained with DAPI (1:1000, D9542, Sigma-Aldrich) for 10 min and rinsed again with PBS before mounted on slides with Fluoromount G mounting medium (00-4958-02, Invitrogen, Thermo Fisher Scientific). Immunofluorescence microscopy was performed with a LSM700 confocal laser microscope (Carl Zeiss, Oberkochen, Germany). Obtained images were analyzed using the Imaris software package (Version 9.3.0, Bitplane, Zürich, CHE). Cell layer thickness was measured in the 3D reconstructed epithelium. Goblet cells, defined by MUC5A expression, as well as basal cells, defined by p63 expression, were manually counted on a 100 µm × 100 µm field of 2D images and counts were interpolated to the total cell culture surface of 1.12 cm^2^. Cell and nuclear volumes were calculated using the in-build function “Cells” of the Imaris software. A 3D model containing each individual cell in the picture based on DAPI (nucleus) and Phalloidin (actin, representing cell membranes) fluorescence was reconstructed with Imaris. To filter out cells which are too big (e.g. merged cells which should be divided into two or more) and cells which are too small (e.g. cell at the edge of the image), the top and bottom 25% of values were assumed to be outliers. The same protocol was applied to all samples.

### Histology

Membranes were sliced out from the insert, cut in halve and put on a filter paper placed in an embedding cassette. Samples were fixed with 7.5% PFA for several hours before overnight dehydration in Tissue-Tek (Sakura Finetek, Alphen aan den Rijn, Netherlands). Afterwards, samples were embedded in paraffin, cut into 1.5 μm sections and stained with hematoxylin and eosin (H&E). The sections were examined by light microscopy.

### Transmission electron microscopy

ALI cultures on inserts were fixed with 2.5% glutaraldehyde and 2% PFA at 4 °C overnight, trimmed to size and incubated in 1% OsO4 at RT. Dehydration in an ascending ethanol series and embedding in Epon resin was carried out according to standard protocols. Lead citrate and uranyl acetate were used to contrast 70 nm ultra-thin sections. Images were taken with a FEI Tecnai20 electron microscope equipped with a 4K Eagle-CCD camera and processed with Adobe Photoshop.

### High-speed video microscopy analysis

ALI cultures were suspended in Medium (Medium199, Gibco) and transferred to glass chamber slides for assessment of ciliary activity by high-speed video microscopy (Th4-200, Olympus) at 37 °C. Ciliated cells were recorded at a rate of 300 frames per second (fps) using a high-speed camera. Beat pattern and frequency were analyzed from 3–5 different cell clusters obtained per sample.

### TEER measurements

TEER measurements were conducted at 28 days and 50 days, respectively. All measurements were performed using a standardized protocol to maintain highest quality and reduce measurement bias. Inserts were assessed within 5 minutes after removal from the incubator to minimize temperature effects. Electrodes were equilibrated and sterilized according to the manufacturer’s instructions. 200 μl of PC-ALI medium were added into the apical chamber. The sample resistance was calculated by subtracting the blank value (cell-free insert) from the total sample resistance. All measurements were conducted with EVOM2 voltohmeter (World Precision Instruments, Sarasota, FL, USA).

### Statistical analysis

Continuous data are summarized as mean and standard deviation or as median and range. Categorical data are summarized as frequencies and percentages. Normal distribution was assessed using Shapiro-Wilk test. Normally distributed data were analyzed using the Student’s *t-*test and data with non-Gaussian distribution were analyzed using the Wilcoxon rank-sum test. Categorical variables were analyzed using Fisher’s Exact test. Statistical significance was defined as *P* < 0.05. Analysis was performed using R 3.6.1 (R core Team, 2019, https://www.R-project.org).

## Supplementary information

Supplementary Video.

Supplementary Video.

Supplementary Information.

## Data Availability

The datasets generated during the current study are available from the corresponding author on request.

## References

[1] Meissner HC (2016). Viral Bronchiolitis in Children. N Engl J Med.

[2] Nair H (2010). Global burden of acute lower respiratory infections due to respiratory syncytial virus in young children: a systematic review and meta-analysis. Lancet.

[3] Collins PL, Melero JA (2011). Progress in understanding and controlling respiratory syncytial virus: still crazy after all these years. Virus Res.

[4] Gern JE, Busse WW (2002). Relationship of viral infections to wheezing illnesses and asthma. Nat Rev Immunol.

[5] Busse WW, Lemanske RF, Gern JE (2010). Role of viral respiratory infections in asthma and asthma exacerbations. Lancet.

[6] Al-Sayed AA, Agu RU, Massoud E (2017). Models for the study of nasal and sinus physiology in health and disease: A review of the literature. Laryngoscope Investig Otolaryngol.

[7] Miller D (2013). Culture of airway epithelial cells from neonates sampled within 48-hours of birth. PLoS One.

[8] Groves HE, Guo-Parke H, Broadbent L, Shields MD, Power UF (2018). Characterisation of morphological differences in well-differentiated nasal epithelial cell cultures from preterm and term infants at birth and one-year. PLoS One.

[9] Jing JC, Chen JJ, Chou L, Wong BJF, Chen Z (2017). Visualization and Detection of Ciliary Beating Pattern and Frequency in the Upper Airway using Phase Resolved Doppler Optical Coherence Tomography. Scientific Reports.

[10] Muller L, Brighton LE, Carson JL, Fischer WA, Jaspers I (2013). Culturing of human nasal epithelial cells at the air liquid interface. J Vis Exp.

[11] Mao H, Wang Y, Yuan W, Wong LB (2009). Ciliogenesis in cryopreserved mammalian tracheal epithelial cells cultured at the air-liquid interface. Cryobiology.

[12] Rayner RE, Makena P, Prasad GL, Cormet-Boyaka E (2019). Optimization of Normal Human Bronchial Epithelial (NHBE) Cell 3D Cultures for *in vitro* Lung Model Studies. Scientific Reports.

[13] Guo-Parke H (2013). Relative respiratory syncytial virus cytopathogenesis in upper and lower respiratory tract epithelium. Am J Respir Crit Care Med.

[14] Schneider D (2012). Neonatal rhinovirus infection induces mucous metaplasia and airways hyperresponsiveness. J Immunol.

[15] Stokes KL (2013). The respiratory syncytial virus fusion protein and neutrophils mediate the airway mucin response to pathogenic respiratory syncytial virus infection. J Virol.

[16] Alves MP (2016). Comparison of innate immune responses towards rhinovirus infection of primary nasal and bronchial epithelial cells. Respirology.

[17] McDougall CM (2008). Nasal epithelial cells as surrogates for bronchial epithelial cells in airway inflammation studies. American journal of respiratory cell and molecular biology.

[18] McLellan K, Shields M, Power U, Turner S (2015). Primary airway epithelial cell culture and asthma in children–lessons learnt and yet to come. Pediatric Pulmonology.

[19] Mihaylova VT (2018). Regional Differences in Airway Epithelial Cells Reveal Tradeoff between Defense against Oxidative Stress and Defense against Rhinovirus. Cell Rep.

[20] Thavagnanam S (2014). Nasal Epithelial Cells Can Act as a Physiological Surrogate for Paediatric Asthma Studies. Plos One.

[21] Ji J (2017). Development of Combining of Human Bronchial Mucosa Models with XposeALI(R) for Exposure of Air Pollution Nanoparticles. PLoS One.

