# Supplementary material for: An optimized, robust and reproducible protocol to generate well-differentiated primary nasal epithelial models from extremely premature infants
Source: Sci Rep. 2019 Dec 27;9:20069. doi: 10.1038/s41598-019-56737-9 (PMC6934534; doi:10.1038/s41598-019-56737-9)
Supplement: Supplementary file 3 — Supplementary Information. [file 41598_2019_56737_MOESM3_ESM.pdf]

MEDICAL UNIVERSITY OF VIENNA

Karolinska Institutet

An optimized, robust and reproducible protocol to generate well-differentiated primary nasal epithelial models from extremely premature infants

Anke Martens, Gabriele Amann, Katy Schmidt, René Gaupmann, Bianca Böhm, Eleonora Dehlink, Zsolt Szépfalusi, Elisabeth Förster-Waldl, Angelika Berger, Nanna Fyhrquist, Harri Alenius, Lukas Wisgrill

Supplementary Files

S1: MATERIAL, ISOLATION AND CULTURING PROTOCOL

TABLE OF CONTENTS

Introduction 3

1 Protocols 3

1.1 Nasal Brush Biopsy 3

1.1.1 Preparation 3

1.1.2 Sampling 3

1.1.3 Day 1 4

1.1.4 Maintenance 5

1.2 Expansion Phase 5

1.2.1 Preparation 5

1.2.2 Procedure 6

1.2.3 Day 1 6

1.2.4 Following Days 6

1.3 Further Propagation 7

1.4 Cryopreservation 7

1.4.1 Preparation 7

1.4.2 Procedure 7

1.5 Thawing 8

1.5.1 Preparation 8

1.5.2 Procedure 8

1.6 Differentiation at Air-Liquid Interface 9

1.6.1 Expansion Phase 9

1.6.2 Establishing the Air-Liquid Interface 10

1.6.3 Differentiation Phase 10

2 Composites 11

Coating Buffer 11

RPMI 1640 Medium 11

PneumaCult-Ex Plus Medium 11

PneumaCult Ex Plus ++ Medium 12

PneumaClut ALI Medium 12

3 Materials 13

INTRODUCTION

To generate well-differentiated pNECs models a fully equipped cell culture laboratory is required. Including a biological safety cabinet, a refrigerated centrifuge and a CO_2 incubator with the following settings:

Temperature:	37°C
CO_2 Concentration:	5%
Humidity:	> 95%

1 PROTOCOLS

1.1 NASAL BRUSH BIOPSY

1.1.1 Preparation

- Coat 3 wells (for adults) or 2 wells (for preterm infants) of a 12-well plate with 500 µl coating buffer per well. Incubate the plate for 30 min at 37°C in the incubator, aspirate the coating buffer and rinse each well with 500 µl PBS.
- Prepare 8 ml transport medium: RPMI 1640 medium supplemented with 1% Antibiotic-Antimycotic and 0.1% Gentamicin (50 mg/ml).

1.1.2 Sampling

1. To obtain pNECs, carefully insert a cytobrush – moistened with sterile NaCl 0,9% - into the nostril and rotate it gently several times. Use a new cytobrush for each nostril.
2. Transfer both brushes into a 15 ml conical tube, containing 8 ml transport medium and detach mucus and cells by gently swirling from the brushes.

3. Discard the cytobrushes and place the conical tube on ice for transport.
4. Centrifuge (4°C, 400x g, 5 min) and aspirate the supernatant.
5. Resolve the pellet in 1ml PneumaCult-Ex Plus medium (Ex-P).
6. Add 10 µl DNase I and mix thoroughly, then incubate for 20 min at room temperature.
7. Centrifuge (4°C, 400x g, 5 min) and do NOT aspirate the supernatant.
8. Add 90 µl PneumaCult Ex Plus ++ medium (Ex-P ++) to each coated well of the 12-well plate (2 wells for adults, 1 well for preterms). (Keep the remaining well dry until day 1.)
9. For preterm pNECs: Aspirate the pellet and transfer it to prepared well while trying to maintain its integrity.

   For adult pNECs: Divide the pellet evenly over both wells but try to keep the cells clustered.
10. If the samples are very mucous, add more Ex-P ++ medium until the pellets are covered with medium.
11. Incubate the cells for 2 hours at 37°C, then check if there is still enough medium, otherwise add another 50 µl Ex-P ++. Leave the cells for 24 hrs in the incubator.

1.1.3 Day 1

1. Examine cells by light microscopy 24 hrs post-seeding, check especially for contaminations (bacteria, fungi).
2. Aspirate the medium from all wells and collect it in a 0.5 ml sterile Eppendorf tube.
3. Centrifuge the tube (4°C, 400x g, 5 min).
4. In the meantime: Add 250 µl Ex-P ++ and 250 µl Ex-P medium to each well from day 0.
5. Use the remaining collagen coated well from day 0 and the non-adherent cells pelleted in the Eppendorf tube to repeat steps 7 – 9 (section 1.1.2).

1.1.4 Maintenance

Examine cells daily by light microscopy to observe their expansion.

Day 2 - 7:

1. Remove the medium.
2. Add the appropriate amount of medium per well:

	PneumaCult-Ex Plus ++ / well	PneumaCult-Ex Plus / well
24 h post-seeding	250 µl	250 µl
48 h post-seeding	125 µl	375 µl
72 h post-seeding & following days	73 µl	427 µl

Cells can be transferred to tissue culture flasks for expansion when they reached 70 – 80% confluency (usually after 7 days). If by day 4 no cells / cell clusters are detectable, the cultivation of the sample can be considered unsuccessful.

1.2 EXPANSION PHASE

One confluent well generally yields two T25 flasks, if the well is less than 70% confluent (after approx. 10 days) use only one T25.

1.2.1 Preparation

- Coat the tissue culture flasks with 3 ml (T25) or 4 ml (T75) coating buffer. Incubate them for 30 min at 37°C in the incubator, aspirate the coating buffer and rinse the flasks with 3 ml or 4 ml PBS.

- Warm the required amount of 0.25% Trypsin-EDTA (500 µl trypsin/ well) to approx. 37°C in a water bath.
- Prepare the appropriate amount of Soy Bean Trypsin Inhibitor (SBTI): 1 ml SBTI (1 mg/ml) inactivates 1 ml 0.25% trypsin.

1.2.2 Procedure

1. Aspirate the medium and add 500 µl trypsin to each well.
2. Incubate the 12-well plate for 2 - 3min at 37°C in the incubator until the cells are detached. Rinse the wells with the trypsin solution to remove remaining adherent cells and transfer the cell suspension into a conical tube containing SBTI.
3. Use 1 ml PBS in total to rinse the wells once more and add it to the tube containing the cell suspension.
4. Centrifuge (4°C, 400x g, 5 min) and aspirate the supernatant.
5. Resuspend the cells in 1 ml Ex-P medium.
6. Add 3 ml (T25) or 5 ml (T75) Ex-P medium to each tissue culture flask.
7. Add the cell suspension evenly to the flasks and place them in the incubator.

1.2.3 Day 1

Examine the cells microscopically 24 hrs post-seeding, if most of the cells are adherent remove the medium from the flask and add 5 ml (T25)/ 10 ml (T75) fresh Ex-P medium. If the majority of cells is still unattached, add only 3 ml (T25) or 5 ml (T75) fresh Ex-P to the remaining medium.

1.2.4 Following Days

Medium needs to be changed every 1 – 2 days, depending on the confluency of cells and the pH of the medium.

Once the cells have reached 80-90% confluency, which usually takes 5 – 8 days, they can be further propagated, cryopreserved or used to establish the air-liquid interface (ALI) cell culture.

1.3 FURTHER PROPAGATION

One confluent T25 flask yields two T75 flasks, and one T75 can be transferred to four T75 flasks.

For further propagation use protocol 1.2 from above, but with few adaptions in section 1.2.2:

- Step 1: Use 3 ml (T25) or 4 ml (T75) 0.25% trypsin instead.
- Step 3: Use 1 ml (T25) or 2 ml (T75) PBS instead.

1.4 CRYOPRESERVATION

1.4.1 Preparation

- Warm 3 ml (T25) / 4 ml (T75) 0.25% Trypsin-EDTA to approx. 37°C in a water bath.
- Prepare the appropriate amount of SBTI: 1 ml SBTI (1 mg/ml) inactivates 1 ml 0.25% trypsin.

1.4.2 Procedure

1. Remove the medium from the flask and rinse with 3 ml (T25) or 4 ml (T75) PBS.
2. Aspirate the PBS and add trypsin.
3. Incubate the flask for 2 – 3 min at 37°C until the cells are detached. Rinse the wells with the trypsin solution to remove remaining adherent cells and transfer the cell suspension into a conical tube containing SBTI.
4. Use 1 ml (T25)/ 2 ml (T75) PBS to rinse the flask once more and add it to the tube containing the cell suspension.

5. Centrifuge (4°C, 400x g, 5 min) and aspirate the supernatant.
6. Resuspend the cells in 1 ml Ex-P medium.
7. Use 10 µl cell suspension and 90 µl Trypan Blue to count the cells with a hemocytometer.
8. Centrifuge the remaining cell suspension (4°C, 400x g, 5 min) and aspirate the supernatant.
9. Dilute the cells in the appropriate amount of BAMBANKER freezing medium. (1 million cells /ml & 1 – 1.5ml cell suspension/ vial)
10. Place the cryogenic vials in a freezing container filled with isopropyl alcohol and store them at –80°C for at least 4 hours. Vials are kept in liquid nitrogen until ALI-differentiation.

1.5 THAWING

Usually one cryovial containing 1 – 1.5 million cells yields one T75 flask.

1.5.1 Preparation

- Coat the tissue culture flask with 4 ml (T75) coating buffer. Incubate it for 30 min at 37°C, aspirate the coating buffer and rinse the flask with 4 ml PBS afterwards.
- Add 10 ml Ex-P medium to the culture flask and prewarm the medium for at least 15 minutes in the incubator.

1.5.2 Procedure

1. Thaw the cells rapidly at 37°C in a water bath.
2. Add the cell suspension to the flask and place it in the incubator.
3. Change the medium within 24 hours after seeding.
4. Continue according to protocol 1.2 section 1.2.3.

1.6 DIFFERENTIATION AT AIR-LIQUID INTERFACE

1.6.1 Expansion Phase

1.6.1.1 Preparation

The following volumes are suitable for 12 mm Transwell® inserts (Corning).

- Coat each insert with 500 µl coating buffer. Incubate the plate for 30 min at 37°C in the incubator, aspirate the coating buffer and rinse each insert with 500 µl PBS.
- Warm 3 ml (T25) / 4 ml (T75) 0.25% Trypsin-EDTA to approx. 37°C in a water bath.
- Prepare the appropriate amount of SBTI: 1 ml SBTI (1 mg/ml) inactivates 1 ml 0.25% trypsin.

1.6.1.2 Procedure

1. Aspirate the medium and add 3 ml (T25)/ 4 ml (T75) trypsin to the flask.
2. Incubate the flask for 2 – 3 min at 37°C until the cells are detached. Rinse the wells with the trypsin solution to remove remaining adherent cells and transfer the cell suspension into a conical tube containing SBTI.
3. Use 1 ml (T25)/ 2 ml (T75) PBS to rinse the flask once more and add it to the tube containing the cell suspension.
4. Centrifuge (4°C, 400x g, 5 min) and aspirate the supernatant.
5. Resuspend the cells in 1 ml Ex-P medium.
6. Use 10 µl cell suspension and 90 µl Trypan Blue to count the cells with a hemocytometer.
7. Dilute the cell suspension with Ex-P medium to obtain a seeding density of 300 000 – 500 000 cells/ 200 µl medium.
8. Add 800 µl Ex-P medium to the basal chamber of each tissue culture insert.

9. Add 200 µl cell suspension to the apical chamber of each insert and place the plate in the incubator.

1.6.1.3 Day 1

24 hrs post-seeding, aspirate the medium on the apical side of the insert and add 200 µl fresh Ex-P medium.

1.6.1.4 Following days

During the first four days of the expansion phase the apical medium should be changed daily, afterwards every two days. The medium in the basal chamber can be changed every other day from the beginning.

Monitor the cells closely, as soon as the cell layer is completely confluent, the air-liquid interface (ALI) can be established.

1.6.2 Establishing the Air-Liquid Interface

1. Aspirate the apical and basal medium.
2. Add 800 µl PneumaClut ALI medium (PC-ALI) to the basal chamber only and keep the apical side exposed to the air.

1.6.3 Differentiation Phase

Change the basal medium every other day (e.g. Monday, Wednesday and Friday). Use 1000 µl medium per insert over the weekend.

After one week at ALI rinse the apical surface of the cell layer with PBS once per week to remove mucus:

1. Add 200 µl PBS/ insert to the apical side.
2. Place the plate for 10 min in the incubator.

3. Aspirate the PBS and exchange the medium in the basal chamber.

Observe the differentiation process every other day using light microscopy. Full differentiation is defined by obvious mucus production and clearly visible ciliary beating throughout the insert.

On average, adult pNECs reach full differentiation after 28 days at ALI, while the preterm pNECs require 50 days to fully differentiate.

2 COMPOSITES

Coating Buffer

Coating buffer contains fibronectin (1 mg/ml), bovine serum albumin fraction V (1 mg/ml) and PureCol (1:100) in PBS without Ca^{2+}/Mg^{2+}. Prepare the required volume in PBS shortly before use.

RPMI 1640 Medium

Supplement RPMI 1640 medium with 1% Antibiotic-Antimycotic and 0.1% Gentamicin (50 mg/ml).

PneumaCult-Ex Plus Medium

1. Thaw 50X-Supplement at room temperature.
2. Mix the supplement gently, do not vortex.
3. Add 490 ml Base Medium, 10 ml 50X-Supplement and 0.5 ml Hydrocortisone Stock Solution to a filter flask.
4. Use a vacuum pump to filter the mixture.
5. Mix gently by inverting the bottle.
6. If desired, aliquots can be made and stored at -20°C until needed.

For cell culture use, supplement the medium with 1% Antibiotic-Antimycotic and 0.1% Gentamicin (50 mg/ml). Ex-Plus medium supplemented with antibiotics is stable for about a week at 4°C.

PneumaCult Ex Plus ++ Medium

Supplement PneumaCult-Ex Plus medium with 5% Nu-Serum, 2% Sodium Bicarbonate 7.5% solution, 1% Antibiotic-Antimycotic and 0.1% Gentamicin (50 mg/ml). Ex-Plus ++ medium is stable for about a week at 4°C.

PneumaClut ALI Medium

PneumaClut ALI Complete Base Medium

1. Thaw 10X-Supplement overnight at 2 – 8°C.
2. Mix the supplement gently, do not vortex.
3. Add 450 ml Base Medium and 50 ml 10X-Supplement to the filter flask.
4. Use a vacuum pump to filter the mixture.
5. Mix gently by inverting the bottle.
6. Prepare aliquots of 45 ml medium each and store them at -20°C until needed.

PneumaClut ALI Maintenance Medium

For cell culture use, add 1% Maintenance Supplement, 0.5% Hydrocortisone Stock Solution and 0.2% Heparin Solution to the PC-ALI Complete Base Medium. PC-ALI Maintenance medium is stable for two weeks at 4°C.

3 MATERIALS

Name	Company	Catalog Number	Comments
RPMI 1640 Medium (with L-Glutamine)	Gibco	21875034	
PneumaCult-Ex-Plus Medium	Stemcell	5040	
Pneumacult-ALI Medium	Stemcell	5001	
BAMBANKER Serum-Free Cell Freezing Medium	BAMBANKER	302-14681	Use as it is.
Antibiotic-Antimycotic (100x)	Gibco	15240062	Use as it is.
Gentamycin	Gibco	15750060	Use as it is.
NuSerum	BD Biosciences	355104	Use as it is.
Sodium Bicarbonate 7.5% Solution	Gibco	25080094	Use as it is.
Heprain Solution	Stemcell	7980	Use as it is.
Hydrocortisone Stock Solution	Stemcell	7925	Use as it is.
DNAse 1, 100 mg	Sigma-Aldrich	DN25	Prepare a concentration of 1.5 mg/ml; dilute in PBS with Ca2+/Mg2+.

Fibronectin, human	Gibco	33016015	1 mg/ ml
Bovine Serum Albumin	Sigma	A8806	1 mg/ ml in PBS
PureCol	AdvancedBioMatrix	5005-B	Use as it is.
Trypsin-EDTA (0.25%), Phenol Red	Gibco	25200056	Use as it is.
Soy Bean Trypsin Inhibitor (SBTI), 100 mg	Sigma-Aldrich	T6522	Dissolve in PBS to a concentration of 1 mg/ml.
Transwell Polyester Membrane Cell Culture Inserts; 12 mm	Corning	3460	
Medscand Cytobrush Plus Endocervical Sampler	Cooper Surgical	C0012	
Dulbecco's Phosphate Buffered Salines (without Ca2+/Mg2+)	Gibco	14190094	
Dulbecco's Phosphate Buffered Salines (with Ca2+/Mg2+)	Gibco	14040141	
Costar 12-well Culture Plates	Corning	3512	

Cell Culture Flasks 25 cm²	TPP	90026	
Cell Culture Flasks 75 cm²	Corning	430641U	
Filter flasks (500 ml)	Corning	431097	
SafeSeal-Tips Professional, Sterile, 1000 µl/ 100 µl/ 10 µl	Biozym	770400X/ 770100X/ 770010X	
Falcon Disposable Polystyrene Serological Pipets, Sterile, 10 ml/ 25 ml	Fisher Scientific	10282371/ 10701942	
Falcon Conical Centrifuge Tubes 50 ml / 15 ml	Fisher Scientific	10788561/ 10773501	
Disposable Glass Pasteur Pipettes, 230 mm, 250/pk	VWR	612-1702	To remove medium from ALI cultures; Use a new pipette for each plate, additionally for apical and basal chambers a new one is required.
Eppendorf Safe-Lock Tubes, 1.5 ml/ 0.5 ml	Eppendorf	0030121589/ 0030121570	
Trypan Blue Solution, 0.4%	Gibco	15250061	Use as it is.

Nunc Biobanking and Cell Culture Cryogenic Tubes, 1.8 ml	Thermo Scientific	368632	
Further cell culture material	ice, pipettes, gloves, lab coat		

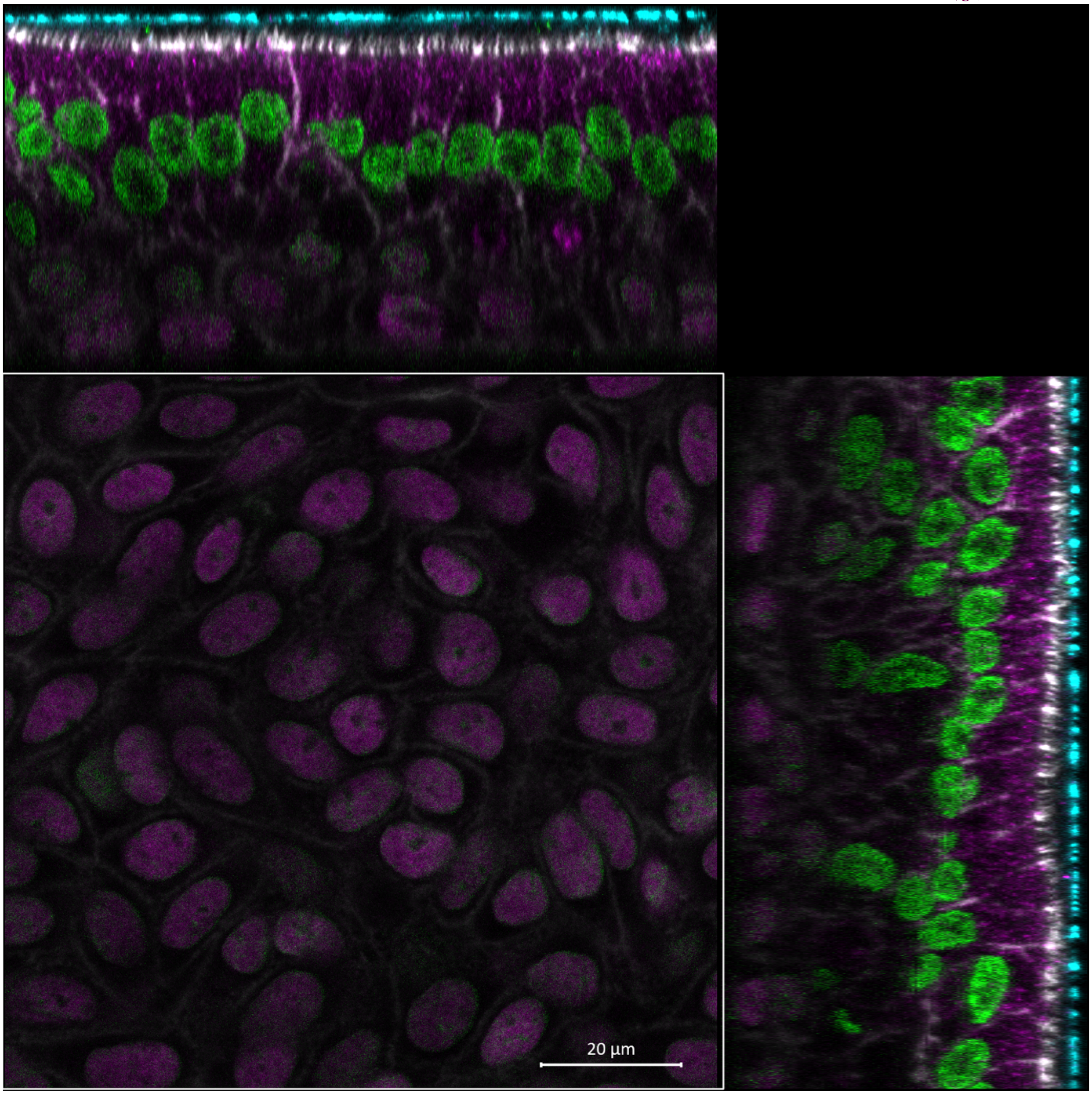

Supplementary Figure 1. Representative image of p63 staining of an adult sample. Cells were stained with p63 (magenta), DAPI (green), α-tubulin (cyan) and Phalloidin (white) and imaged on an LSM700 (Carl Zeiss) using the z-stack function. The basal cells are clearly visualized on the bottom of the cell layers. Scale bar: 20 µm.
